# Vitamin D Deficiency May Not Be an Independent Risk Factor for Peripheral Arterial Disease in Middle-Aged and Elderly Patients with Type 2 Diabetes in China

**DOI:** 10.1155/2020/8854717

**Published:** 2020-11-25

**Authors:** Yan Wang, Tongbao Feng, Hongxing Zhou, Kefeng Lu, Yang Bai, Ping Zhang

**Affiliations:** Department of Clinical Laboratory, The Affiliated Changzhou No.2 People's Hospital of Nanjing Medical University, Changzhou, China

## Abstract

**Background:**

Vitamin D deficiency can lead to the increased severity and prevalence of metabolic disorders. However, the relationship between levels of 25-hydroxyvitamin D (25(OH)D) and peripheral arterial disease (PAD) is controversial. Therefore, the purpose of our study was to explore the relationship between 25(OH)D levels and PAD in middle-aged and elderly type 2 diabetes mellitus (T2DM) patients in China.

**Methods:**

In this study, a total of 183 patients with T2DM were enrolled and categorized into groups with or without PAD. Clinical and biochemical parameters were assessed, and a Pearson analysis was used to identify a possible association between levels of 25(OH)D and glycated hemoglobin (HbA1c). Some biochemical parameters were also assessed in the T2DM patients with PAD according to vitamin D status. Interactions were also explored among HbA1c control, 25(OH)D levels, and PAD. The possible risk factors for PAD were measured by multivariable logistic regression analyses.

**Results:**

Firstly, the parameters including age, HbA1c, and disease duration between T2DM and T2DM+PAD groups showed significantly different. In addition, the frequency of smoking in the group of T2DM patients was significantly less than that in the T2DM patients with the PAD group, while the frequency of well-controlled HbA1c in the patients with T2DM was significantly higher. There is a trend that the levels of 25(OH)D and HbA1c are correlated, but no interactions among vitamin D deficiency, HbA1c control, and PAD were found. However, HbA1c significantly differed between groups with vitamin D deficiency and insufficiency in the T2DM patients with PAD. According to the multivariate logistic regression analyses, the PAD risk factors of T2DM patients were family history of diabetes, smoking, age, disease duration, HbA1c, and LDL.

**Conclusions:**

The findings demonstrate that the deficiency of vitamin D level is not related to PAD, but HbA1c may be linked to the presence of PAD in middle-aged and elderly patients with T2DM in China.

## 1. Introduction

Peripheral arterial disease (PAD), a prevalent diabetic complication, although most PAD patients are asymptomatic, also can raise the risk of morbidity of these patients [1]. PAD can reduce blood flow and obstruct arteries due to atherosclerotic plaques [2], which can cause an increased risk of limb and cardiovascular disease [3, 4]. A review showed a higher incidence of PAD in people over the age of 65 years [5]. Therefore, the early designation, assessment, and treatment of T2DM patients with higher risks of PAD are critical and warranted.

It is extremely clear that vitamin D exerts a significant effect on the processes of bone mineral metabolism and maintenance of calcium homeostasis. Recent evidence has also shown the vascular endothelial damage [6] and the prevalence of diabetes [7] caused by the deficiency of vitamin D. Vitamin D deficiency is a common condition in the world. Therefore, multiple studies have uncovered the relationship between levels of 25-hydroxyvitamin D (25(OH)D) and PAD; however, the results are inconsistent. While some studies have demonstrated that deficiency of vitamin D and PAD showed a correlation [8, 9], another found no association between levels of 25(OH)D and PAD popularity and severity [10].

To date, evidence is limited on indicating the relationships between 25(OH)D levels and PAD in middle-aged and elderly patients with T2DM in China. Therefore, the objective of this study was to uncover the possible underlying effects of 25(OH)D on PAD in middle-aged and elderly patients with T2DM in China.

## 2. Materials and Methods

### 2.1. Patients and Samples

183 T2DM-diagnosed patients were collected in our study from the Department of Endocrinology at Changzhou No.2 People's Hospital. According to the World Health Organization diagnostic criteria, T2DM was diagnosed if the glucose value of a fasting plasma is ≥7.0 mmol/L or a 2 h value during an oral test of glucose tolerance is ≥11.1 mmol/L [11]. All the 183 T2DM patients, 108 males and 75 females, were separated into T2DM with (T2DM+PAD) or without PAD (T2DM) group. Patients with hepatic disease, acute illnesses, chronic kidney disease, cancer present, a disease affecting vitamin D metabolism, or type 1 diabetes mellitus were excluded. In addition, those with drugs interfering with vitamin D status or calcium level, or with glucocorticoids or calcitonin in the past 3 months, were not included. Patients aged <40 or >65 years were also excluded.

Relevant parameters including age, sex, disease duration, hypertension, smoking habits, and family history of diabetes were collected by face-to-face interviews. Hypertension was diagnosed with blood pressure (BP) ≥ 140/90 mmHg or administration of antihypertensive drugs. Patients were classified as smokers or nonsmokers, with subjects who had never had cigarettes in their life defined as nonsmokers. Smokers were defined as people with the habit of smoking including those who quit forever. Body height (in meters, m) and weight (in kilograms, kg) were checked and documented, and the calculation of weight (kg) divided by the square of body height (m) was used as an assessment of corrected weight body mass index (BMI). The season when blood samples were obtained was recorded for all patients. As this study was conducted in Changzhou (latitude 31.78°S), a city in the south of China, June to October was defined as the summer-autumn season, and November to May of the following year was defined as the winter-spring season. All patients had free exposure to sunlight. This study was carried out with the consent of the Committee of Ethics at Changzhou No.2 People's Hospital. Each participant was notified and enrolled in the study with written consent forms.

### 2.2. Diagnosis of Peripheral Arterial Disease (PAD)

As a measurement to diagnose PAD, the ankle-brachial blood pressure index (ABI) was calculated as the ankle-to-arm systolic blood pressure ratio [12]. The blood pressures of both arm and ankle were tested using a device equipped with ABI output (VaSera VS-1000, Japan). A value of ABI < 0.9 indicated the diagnosis of PAD [13, 14]. Patients were also classified as having PAD during angioplasty examination, as in our previous study [15].

### 2.3. Biochemical Measurements

All the fasting blood samples of enrolled patients were collected the next morning and processed within 1 h to test the biochemical variables. Triglycerides (TG), low-density lipoprotein (LDL), high-density lipoprotein (HDL), total cholesterol (TC), lipoprotein B (ApoB), lipoprotein A1 (ApoA1), lipoprotein A (LPA), serum creatinine, phosphate, calcium, and uric acid were tested using automatic analyzers (Cobas 8000, Germany). Parathormone (PTH) levels were measured by radioimmunoassay (Cobas e602, Germany). The high-performance liquid chromatography (HPLC) method was used for the determination of glycated hemoglobin (HbA1c) values (Tosoh G8, Japan). Glycemic control values were classified as poorly controlled (HbA1c ≥ 7%) and well controlled (HbA1c < 7%) [16]. The levels of serum 25(OH)D were detected by an autoanalyzer (Cobas 8000, Germany) with an electrochemiluminescence immunoassay. In this study, the 25(OH)D value < 50 nmol/L was regarded as deficiency of vitamin D, vitamin D sufficiency was defined as 25(OH)D > 75 nmol/L, and 25(OH)D levels between 50 and 75 nmol/L were considered as vitamin D insufficiency [17, 18].

### 2.4. Statistical Analysis

Data collected and analyzed in this study were presented as the mean ± SD. All analyses to measure the statistical significance were carried out using SPSS 16.0 (SPSS Inc., Chicago, USA). Differences of clinical parameters including age, BMI, disease duration, HbA1c, TG, HDL, TC, LDL, ApoA1, ApoB, LPA, serum calcium, serum phosphate, serum creatinine, serum uric acid, 25(OH)D, and PTH values between the two groups were measured by independent sample Student's *t* tests. And analyses of classified data such as hypertension, sex, smoking habits, and family history of diabetes were conducted by a chi-squared test or Fisher's exact test. The variables among the three different-levels-of-25(OH)D groups in the T2DM patients with PAD were compared by an analysis of variance (ANOVA). LSD-*t* is used when the variances between groups are assumed. An analysis of multivariate logistic regression was performed for assessments of the PAD risk factors, and the value was represented as an adjusted odds ratio (OR) with 95% confidence intervals (CI). The value of *P* < 0.05 was of statistical significance.

## 3. Results

### 3.1. Clinical Characteristics of the Patients Enrolled in the Study

The clinical data for the T2DM patients are displayed in Table 1. Of the T2DM patients with PAD, 74 (63.2%) were male, 48 (41.0%) had a diabetic family history, and 53 (45.3%) had hypertension. Differences between the two groups for HbA1c, smoking habits, age, and disease duration were measured significantly. In addition, patients with well-controlled HbA1c showed a higher proportion in the T2DM group (*P* ≤ 0.001). No significant difference was found in the serum 25(OH)D levels in T2DM patients with or without PAD (54.46 ± 11.87 nmol/L vs. 50.84 ± 12.31 nmol/L, *P* = 0.055). The frequency of vitamin D deficiency also showed no difference (23, 34.8% vs. 58, 49.6%, *P* = 0.054). No differences in other clinical data between the two groups showed statistically significant.

### 3.2. Correlation between Serum 25(OH)D Levels and HbA1c

**Figure 1:**
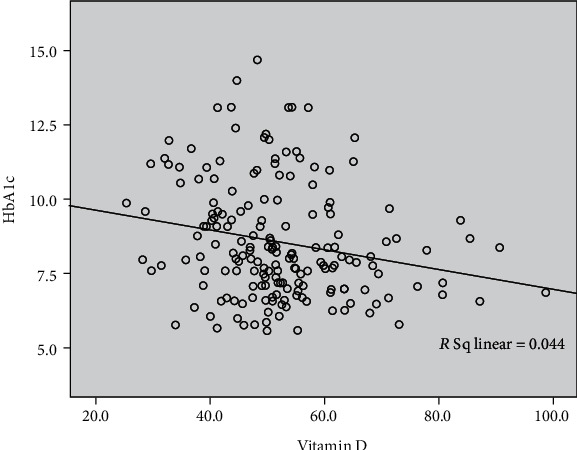
The relationship between serum levels of vitamin D and glycated hemoglobin (HbA1c) in all analyzed patients.

The dot chart displayed in Figure 1 shows there is a trend that the levels of 25(OH)D and HbA1c are correlated, with a correlation coefficient of −0.209, despite *P* < 0.05.

### 3.3. Association of Levels of 25(OH)D with Lipid Profiles and HbA1c

The association between 25(OH)D level, the lipid profiles, and HbA1c in the T2MD+PAD group is displayed in Table 2. Compared to the vitamin D sufficiency group, the levels of TC and LDL in the group of vitamin D deficiency were higher and HDL levels were lower, but no significant differences were found. However, the difference of HbA1c levels between the two groups of vitamin D deficiency and insufficiency was significant.

### 3.4. Serum 25(OH)D Levels, HbA1c, and Their Interaction with PAD

**Figure 2:**
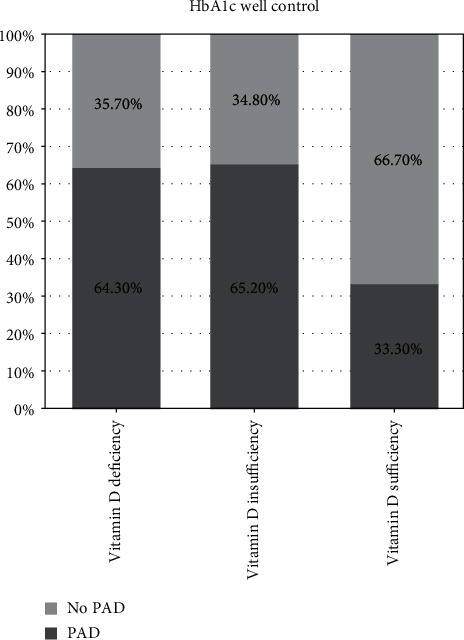
Distribution of peripheral arterial disease (PAD) in different vitamin D status groups of patients with well-controlled glycemia. HbA1c: glycated hemoglobin; well-controlled HbA1c defined as HbA1c < 7%. The levels of vitamin D was represented as 25-hydroxyvitamin-D (25(OH)D) levels: deficiency, 25(OH)D value < 50 nmol/L; insufficiency, 25(OH)D value ≥ 50 and <75 nmol/L; sufficiency, 25(OH)D value ≥ 75 nmol/L.

**Figure 3:**
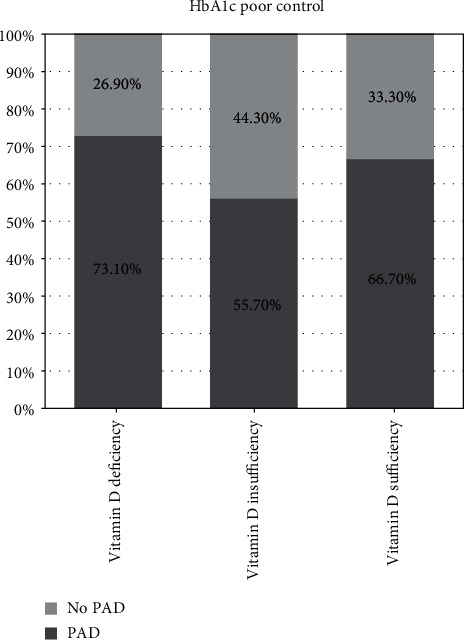
Distribution of peripheral arterial disease (PAD) in glycemic poorly controlled patients and vitamin D status of deficiency, insufficiency, or sufficiency. HbA1c: glycated hemoglobin; well-controlled HbA1c defined as HbA1c < 7%.

Figure 2 displays a stacked bar chart showing no significant difference between three groups based on the levels of vitamin D in glycemic well-controlled patients. Additionally, the proportion of patients with PAD in patients with sufficiency of vitamin D was lower among the three groups, but the difference was not statistically significant.  Figure 3 is also a stacked bar chart showing no significant difference when comparing the three groups in the poorly controlled glycemia patients. The proportion of PAD patients was similar between those three groups.

### 3.5. Association between Clinical Variables with the Risk of PAD

The clinical variables associated with PAD were analyzed by an analysis of multivariate logistic regression. The PAD presence (1 = PAD, 0 = non‐PAD) was a dependent variable considering all subjects as a whole. Sex (1 = male, 2 = female), diabetic family history (1 = yes, 0 = no), smoking (1 = smoker, 0 = nonsmoker), hypertension (1 = hypertension, 0 = nonhypertension), BMI, disease duration, HbA1c, 25(OH)D, TC, HDL, TG, LDL, ApoB, ApoA1, and LPA were regarded as covariates. The results displayed in Table 3 show that age (*P* = 0.011), diabetic family history (*P* = 0.043), disease duration (*P* = 0.001), smoking (*P* = 0.006), LDL (*P* = 0.031), and HbA1c (*P* = 0.016) were significant factors for prediction of the PAD presence in T2DM patients. Multivariate logistic regression further verified that 25(OH)D was irrelevant to PAD in T2DM patients (*P* = 0.116).

## 4. Discussion

Our results indicate that deficiency of vitamin D was not correlated to PAD. However, HbA1c may be correlated with the appearance of PAD in middle-aged and elderly T2DM patients in China. In our study, we refer PAD to limb condition with arterial occlusion [19]. The occurrence and progression of PAD are linked to many diseases or factors such as ethnicity, abnormal lipid metabolism, and diabetes [20]. Besides the established risk factors including age [21], smoking [20], and dyslipidemia [22] for PAD, HbA1c was found likely to be a PAD independent risk factor, which is consistent with our previous study [15]. HbA1c, the important factor of detecting glycemic control, is produced by the nonenzymatic glycation reaction in the blood based on hemoglobin and glucose. Additionally, glucose, protein, and lipids under the reactions of nonenzymatic glycosylation can produce advanced glycation end products (AGEs) [23]. Thus, HbA1c shows a significant association with AGEs, which can accelerate the progression of PAD in several ways [24–27].

A recent Chinese study has indicated that the deficiency of vitamin D is associated with PAD in T2DM patients after adjustment for various risk factors [9]. Another study has also confirmed that deficiency of vitamin D, as an independent factor, can increase the risks of PAD incidence [28]. One possible explanation may be that a normal vitamin D level can prevent foam cell formation, while vitamin D deficiency may promote the progression of atherosclerosis [29]. In this study, however, we found no association between the levels of 25(OH)D and PAD in middle-aged and elderly T2DM patients, possibly because the number of PAD patients is relatively small. A second possible reason may be due to age differences, as our patients were younger (limited to middle-aged and elderly patients) than the populations in the other studies. The vitamin D receptor expression level descends linearly with age [30], suggesting that extremely high levels of 25(OH)D would probably be a necessity to the elderly to prevent atherosclerotic processes [31]. In addition, our diagnostic criterion for PAD differed from the other studies, as we also included patients identified during angioplasty examinations.

In our study, there is a trend that the levels of serum 25(OH)D are correlated with HbA1c, which significantly differed between patients with insufficient and deficient vitamin D in T2DM patients with PAD. This suggests that active glucose control not only is a benefit to the PAD prevention and therapy but also may be beneficial for the increase of 25(OH)D.

In terms of limitations of considering only Chinese people in the present study, the results generalizing to other ethnic people can probably be not appropriate due to racial different levels of 25(OH)D [32]. In addition, data on drug therapy were not collected and analyzed which is warranted to be done in the future in-depth studies.

## 5. Conclusions

In conclusion, findings in this study demonstrated that deficiency of vitamin D was not linked to PAD, but HbA1c may be correlated to the occurrence of PAD in middle-aged and elderly patients with T2DM in China. Furthermore, the prevention of PAD incidence and progression should be studied and performed in T2DM patients with clinical characteristics with high risks.

## Figures and Tables

**Table 1:** Clinical and biochemical characteristics of type 2 diabetes mellitus (T2DM) patients with and without peripheral arterial disease (PAD).

Parameters	All participants (*n* = 183)	T2DM (*n* = 66)	T2DM+PAD (*n* = 117)	*P*
Age (years)	55.42 ± 7.20	53.20 ± 7.23	56.68 ± 6.91	0.002^∗^
Male, *n* (%)	108 (59.0%)	34 (51.5%)	74 (63.2%)	0.121
Disease duration (years)	6.90 ± 6.13	4.47 ± 4.70	8.27 ± 6.43	0.001^∗^
Hypertension, *n* (%)	79 (43.2%)	26 (39.4%)	53 (45.3%)	0.439
Smoking, *n* (%)	83 (45.4%)	22 (33.3%)	61 (52.1%)	0.014^∗^
Diabetes, *n* (%)	82 (44.8%)	34 (51.5%)	48 (41.0%)	0.171
BMI (kg/m^2^)	24.95 ± 3.12	24.96 ± 3.16	24.95 ± 3.11	0.971
TG (mmol/L)	1.85 ± 0.99	1.98 ± 1.09	1.77 ± 0.93	0.181
LDL (mmol/L)	2.51 ± 0.72	2.39 ± 0.73	2.58 ± 0.71	0.096
HDL (mmol/L)	1.10 ± 0.31	1.12 ± 0.38	1.10 ± 0.26	0.702
TC (mmol/L)	4.60 ± 0.87	4.53 ± 0.83	4.64 ± 0.89	0.432
ApoB (g/L)	1.05 ± 0.76	0.98 ± 0.21	1.09 ± 0.94	0.348
ApoA1 (g/L)	1.24 ± 0.21	1.25 ± 0.21	1.23 ± 0.20	0.639
LPA (g/L)	0.14 ± 0.15	0.13 ± 0.11	0.15 ± 0.16	0.244
HbA1c (%)	8.54 ± 1.93	8.16 ± 1.64	8.76 ± 2.06	0.034^∗^
25(OH)D (nmol/L)	52.15 ± 12.24	54.46 ± 11.87	50.84 ± 12.31	0.055
Creatinine (*μ*mol/L)	63.17 ± 14.43	62.04 ± 15.15	63.81 ± 14.03	0.426
Phosphate (mmol/L)	1.15 ± 0.17	1.18 ± 0.16	1.13 ± 0.18	0.103
Calcium (mmol/L)	2.24 ± 0.10	2.24 ± 0.09	2.24 ± 0.10	0.999
Serum uric acid (*μ*mol/L)	299.70 ± 71.18	304.73 ± 67.61	296.50 ± 73.25	0.475
PTH (ng/L)	40.03 ± 12.75	38.75 ± 12.69	40.75 ± 12.78	0.308
HbA1c controlled, *n* (%)				0.001^∗^
Yes	40 (21.9%)	25 (37.9%)	15 (12.8%)	
No	143 (78.1%)	41 (62.1%)	102 (87.2%)	
Vitamin D deficiency, *n* (%)				0.054
Yes	81 (44.3%)	23 (34.8%)	58 (49.6%)	
No	102 (55.7%)	43 (65.2%)	59 (50.4%)	
Season, *n* (%)				0.678
Spring-winter	98 (53.6%)	34 (51.5%)	64 (54.7%)	
Summer-autumn	85 (46.4%)	32 (48.5%)	53 (45.3%)	

^∗^ *P* < 0.05. BMI: body mass index; TG: triglycerides; LDL: low-density lipoprotein; HDL: high-density lipoprotein; TC: total cholesterol; ApoB: lipoprotein B; ApoA1: lipoprotein A1; LPA: lipoprotein A; HbA1c: glycated hemoglobin; 25(OH)D: 25-hydroxyvitamin-D; PTH: parathormone; vitamin D deficiency: 25(OH)D < 50 nmol/L; HbA1c controlled: HbA1c < 7%.

**Table 2:** Biochemical parameters according to vitamin D status in T2DM patients with peripheral arterial disease.

Group	Number	TC	HbA1c	LDL	HDL
A	58	4.71 ± 0.90	9.21 ± 2.10	2.64 ± 0.75	1.11 ± 0.27
B	54	4.58 ± 0.91	8.35 ± 2.00	2.53 ± 0.69	1.08 ± 0.25
C	5	4.50 ± 0.45	7.86 ± 0.85	2.39 ± 0.27	1.18 ± 0.18
LSD-*t*, *P*	A vs. C	0.627	0.155	0.444	0.577
	B vs. C	0.852	0.599	0.681	0.438
	A vs. B	0.462	0.028^∗^	0.384	0.586

^∗^ *P* < 0.05. A: deficiency of vitamin D, 25(OH)D level < 50 nmol/L; B: insufficiency of vitamin D, 25(OH)D level < 75 and ≥50 nmol/L; C: sufficiency of vitamin D, 25(OH)D level ≥ 75 nmol/L; 25(OH)D: 25-hydroxyvitamin-D; TC: total cholesterol; HbA1c: glycated hemoglobin; LDL: low-density lipoprotein; HDL: high-density lipoprotein.

**Table 3:** Analyses of multivariate logistic regression for peripheral arterial disease-associated clinical factors in all patients.

Parameters	OR	95% CI	*P*
Age	1.076	1.017–1.138	0.011^∗^
Sex	1.011	0.369–2.776	0.982
Diabetic family history	0.475	0.231–0.977	0.043^∗^
Smoking	2.844	1.349–5.996	0.006^∗^
Disease duration	1.137	1.056–1.225	0.001^∗^
Hypertension	0.880	0.400–1.939	0.752
BMI	1.008	0.874–1.162	0.914
TG	0.763	0.481–1.211	0.251
LDL	1.709	1.052–2.778	0.031^∗^
HDL	0.795	0.143–4.415	0.793
TC	1.123	0.468–2.695	0.796
ApoB	1.290	0.488–3.411	0.608
ApoA1	0.737	0.054–10.053	0.819
LPA	1.036	0.067–12.029	0.980
HbA1c	1.282	1.047–1.570	0.016^∗^
25(OH)D	0.975	0.944–1.006	0.116

^∗^ *P* < 0.05. A multivariate logistic regression analysis was performed to analyze the data. OR: odds ratio; CI: confidence interval; BMI: body mass index; TG: triglycerides; LDL: low-density lipoprotein; HDL: high-density lipoprotein; TC: total cholesterol; ApoB: lipoprotein B; ApoA1: lipoprotein A1; LPA: lipoprotein A; HbA1c: glycated hemoglobin; 25(OH)D: 25-hydroxyvitamin-D.

## Data Availability

Data are not available. All the findings of this study are carried out by analyzing the datasets that appeared in this manuscript.
